# ACC-1 β-Lactamase–producing *Salmonella enterica* Serovar Typhi, India

**DOI:** 10.3201/eid1607.091643

**Published:** 2010-07

**Authors:** Bindiganavile N. Gokul, Godfred A. Menezes, Belgode N. Harish

**Affiliations:** Author affiliations: Sri Devaraj Urs Medical College, Kolar, India (B.N. Gokul, G.A. Menezes);; Fortis Hospitals, Bangalore, India (B.N. Gokul);; Jawaharlal Institute of Postgraduate Medical Education and Research, Pondicherry, India (G.A. Menezes, B.N. Harish)

**Keywords:** Emergence, AmpC, Salmonella enterica serovar Typhi, bacteria, antimicrobial drug resistance, enteric infections, letter

**To the Editor:** Typhoid fever, caused by *Salmonella enterica* serovar Typhi, is a serious form of enteric fever. In 2000, the worldwide number of typhoid cases was estimated to be >21,000,000, and there were >200,000 deaths from this disease (*1*).

Ciprofloxacin is the first-line drug of choice for treatment of patients with typhoid fever, but there has been an increase in strains resistant to ciprofloxacin (*2*) and resistance to third-generation cephalosporins has emerged (*3*). There are sporadic reports of high resistance to ceftriaxone in typhoidal salmonellae (*3**,**4*) in which CTX-M-15 and SHV-12 extended spectrum β-lactamases (ESBLs) have been reported. To date, there are no reports of *AmpC* β-lactamases in typhoidal salmonellae. *AmpC* β-lactamases confer resistance to a broad spectrum of β-lactams, which greatly limits therapeutic options. We investigated an isolate of *S.* Typhi by using serotyping, antimicrobial drug susceptibility testing, PCR screening for β–lactamase genes, and sequence analysis to confirm the identity of the isolate and the β–lactamase gene involved in conferring resistance to this isolate.

The isolate was obtained in Bangalore, India, in August 2009, from the blood of a female patient (14 years of age) who was hospitalized because of signs and symptoms of enteric fever. She had no history of having received antimicrobial drugs. After a blood sample was cultured, the patient was empirically treated with ceftriaxone but did not clinically improve.

Culture yielded gram-negative bacteria after 48 hours. The isolate was identified by standard biochemical methods as *S.* Typhi. Identification was confirmed by using *Salmonella* spp. polyvalent O, O9, and H:d antisera (Murex Biotech, Dartford, UK). Susceptibility to antimicrobial drugs was assessed by using the Kirby-Bauer disk diffusion method according to Clinical and Laboratory Standards Institute guidelines (www.clsi.org). The isolate was resistant to ampicillin, piperacillin, cefoxitin, cefotaxime, ceftazidime, ceftriaxone, aztreonam, amoxicillin/clavulanate, and cefepime. It was susceptible to chloramphenicol, trimethoprim/sulfamethoxazole, nalidixic acid, ciprofloxacin, and meropenem.

Treatment was changed to ciprofloxacin (500 mg every 12 h for 7 d). The patient recovered within 72 hours and was discharged. MICs were determined for ciprofloxacin, gatifloxacin, ofloxacin, ceftazidime, ceftriaxone, and amoxicillin/clavulanate by using the Etest (AB Biodisk, Solna, Sweden) (Table). MIC for ceftriaxone was confirmed by an agar dilution method (www.clsi.org). The isolate was tested for ESBLs by using a method with disks containing ceftazidime (30 μg) and ceftazidime/clavulanate (30 μg/10 μg). The *AmpC* disk test for detection of plasmid-mediated *AmpC* β-lactamase was conducted according to standard methods (*5*).

**Table:** MICs for isolate of *Salmonella enterica* serovar Typhi, Bangalore, India, 2009

Drug	MIC
Amoxicillin/clavulanic acid	>256
Piperacillin/tazobactam	12
Ceftazidime	>256
Cefotaxime	>256
Ceftriaxone	>256
Ciprofloxacin	0.094

PCR screening and sequencing was performed to identify β-lactamase resistance genes *bla*_TEM_, *bla*_SHV_, *bla*_OXA-1_ group, *bla*_CTX-M_, and *AmpC* as described (*6**,**7*). Sequencing of β-lactamase gene amplicons was conducted at the Vector Control Research Centre in Pondicherry, India. The BLASTN program (www.ncbi.nlm.nih.gov/BLAST) was used for database searching. We also used a nested PCR specific for the flagellin gene of *S.* Typhi to confirm identity of the isolate (*8*). The nested PCR amplicon was sequenced to confirm identity of the flagellin (*fliC*) gene of *S.* Typhi. Sequencing of the flagellin gene product was conducted by Cistron Bioscience (Chennai, India).

The isolate was negative for ESBL production. PCR amplification and sequencing showed that the isolate harbored *bla*_TEM-1_ and *bla*_ACC-1._ The isolate was negative by PCR for other β-lactamases tested. TEM-1 is one of the most commonly encountered β-lactamases in the family *Enterobacteriaceae* and can hydrolyze narrow-spectrum penicillins and cephalosporins.

We report ACC-1 *AmpC* β-lactamase in typhoidal salmonellae. *S.* Typhi could have acquired the *AmpC* β- lactamase from drug-resistant bowel flora. After the isolate was found to be highly resistant to ceftriaxone, the change in therapy to ciprofloxacin helped in recovery of the patient without any sequelae.

ACC-1 *AmpC* β-lactamases originated in *Hafnia alvei* and are now found in various members of the family *Enterobacteriaceae* (*9*). The ACC-1 *AmpC* β-lactamases are exceptional in that they do not confer resistance to cephamycins (*10*). Our isolate contained *bla*_TEM-1_ and *bla*_ACC-1_ and was resistant to cefoxitin and cefepime but susceptible to meropenem. Bidet et al. (*9*) reported isolating *Klebsiella pneumoniae* resistant to cefoxitin and cefepime and intermediate resistance to imipenem. Atypical resistance was attributed to ACC-1 β-lactamase production and loss of a 36-kDa major outer membrane protein (*9*). We did not analyze changes in the outer membrane proteins responsible for alteration of permeability.

Continual monitoring of drug resistance patterns is imperative. Antimicrobial drug susceptibility testing should be conducted for clinical isolates, and empirical antimicrobial drug therapy should be changed accordingly. *AmpC* β-lactamase genes will eventually be transferred to typhoidal salmonellae, which may pose a threat to public health. Spread of broad-spectrum β-lactamases would greatly limit therapeutic options and leave only carbapenems and tigecycline as secondary antimicrobial drugs.
